# Goal-Directed Fluid Therapy Using Pulse Pressure Variation in Thoracic Surgery Requiring One-Lung Ventilation: A Randomized Controlled Trial

**DOI:** 10.3390/jcm13185589

**Published:** 2024-09-20

**Authors:** Giovanni Punzo, Giovanna Beccia, Chiara Cambise, Tiziana Iacobucci, Flaminio Sessa, Mauro Sgreccia, Teresa Sacco, Angela Leone, Maria Teresa Congedo, Elisa Meacci, Stefano Margaritora, Liliana Sollazzi, Paola Aceto

**Affiliations:** 1 Department of Emergency, Anesthesiological and Reanimation Sciences, Fondazione Policlinico Universitario Agostino Gemelli IRCCS, 00168 Rome, Italy; giovanni.punzo@policlinicogemelli.it (G.P.); giovanna.beccia@policlinicogemelli.it (G.B.); chiara.cambise@policlinicogemelli.it (C.C.); tiziana.iacobucci@policlinicogemelli.it (T.I.); flaminio.sessa@policlinicogemelli.it (F.S.); mauro.sgreccia@policlinicogemelli.it (M.S.); teresa.sacco@policlinicogemelli.it (T.S.); leone.angela.90@gmail.com (A.L.); liliana.sollazzi@unicatt.it (L.S.); 2 Department of General Thoracic Surgery, Fondazione Policlinico Universitario Agostino Gemelli IRCCS, 00168 Rome, Italy; mariateresa.congedo@policlinicogemelli.it (M.T.C.); elisa.meacci@policlinicogemelli.it (E.M.); stefano.margaritora@policlinicogemelli.it (S.M.); 3 Department of Translational Medicine and Surgery, Università Cattolica del Sacro Cuore, 00168 Rome, Italy; 4 Department of Basic Biotechnological Science, Intensive Care and Peri-Operative Clinics, Università Cattolica del Sacro Cuore, 00168 Rome, Italy

**Keywords:** goal-directed therapy, GDT, pulse pressure variation, PPV, one-lung ventilation, OLV, thoracic surgery, U-VATS

## Abstract

**Background**: Intraoperative fluid management based on pulse pressure variation has shown potential to reduce postoperative pulmonary complications (PPCs) and improve clinical outcomes in various surgical settings. However, its efficacy and safety have not been assessed in patients undergoing thoracic surgery with one-lung ventilation. **Methods**: Patients scheduled for pulmonary lobectomy using uniportal video-assisted thoracic surgery approach were randomly assigned to two groups. In the PPV group, fluid administration was guided by the pulse pressure variation parameter, while in the near-zero group, it was guided by conventional hemodynamic parameters. The primary outcome was the partial pressure of oxygen (PaO_2_)/ fraction of inspired oxygen (FiO_2_) ratio 15 min after extubation. The secondary outcomes included extubation time, the incidence of postoperative pulmonary complications in the first three postoperative days, and the length of hospital stay. **Results**: The PaO_2_/FiO_2_ ratio did not differ between the two groups (364.48 ± 38.06 vs. 359.21 ± 36.95; *p* = 0.51), although patients in the PPV group (*n* = 44) received a larger amount of both crystalloids (1145 ± 470.21 vs. 890 ± 459.31, *p* = 0.01) and colloids (162.5 ± 278.31 vs 18.18 ± 94.68, *p* = 0.002) compared to the near-zero group (*n* = 44). No differences were found in extubation time, type and number of PPCs, and length of hospital stay. **Conclusions**: PPV-guided fluid management in thoracic surgery requiring one-lung ventilation does not improve pulmonary gas exchange as measured by the PaO_2_/FiO_2_ ratio and does not seem to offer clinical benefits. Additionally, it results in increased fluid administration compared to fluid management based on conventional hemodynamic parameters.

## 1. Introduction

Fluid therapy holds significant importance in thoracic surgery, particularly for patients undergoing one-lung ventilation (OLV), as the fluid overload is linked to severe postoperative respiratory complications, including acute lung injury (ALI), acute respiratory distress syndrome (ARDS), pneumonia, and atelectasis [1,2,3,4]. Therefore, current guidelines for the perioperative management of thoracic surgery patients strongly advise against liberal fluid regimens, recommending “moderate” or “restrictive” approaches to avoid fluid overload while ensuring euvolemia [5]. However, determining the precise infusion rate needed to maintain euvolemia without causing volume overload or tissue hypoperfusion is challenging. This challenge has led to varying interpretations of what constitutes “moderate” or “restrictive” fluid management across different centers [6,7,8,9], prompting the exploration of new fluid management strategies in thoracic surgery.

One such strategy, Goal-Directed Therapy (GDT), is currently under significant discussion [10]. GDT uses flow-related hemodynamic parameters, such as stroke volume variation (SVV) and pulse pressure variation (PPV), to guide intraoperative fluid administration, optimize intravascular volume status, and potentially improve clinical outcomes [10,11,12,13,14,15]. In the few studies on thoracic surgery patients requiring OLV, SVV-based GDT protocols have shown increased partial pressure of oxygen (PaO_2_)/ the fraction of inspired oxygen (FiO_2_) ratios, shorter extubation times, and reduced postoperative nausea and vomiting (PONV) compared to traditional fluid management strategies [16,17]. However, SVV monitoring requires specific devices, such as the Vigileo-FloTrac system, which may not be widely available. Additionally, the reliability of SVV in predicting fluid responsiveness in OLV patients, especially with low tidal volumes, has been recently questioned [18].

PPV, another flow-related hemodynamic parameter [19,20], is commonly monitored in most surgical patients due to its integration into nearly all modern anesthesia machines. It is cost-effective, and its use in high-risk surgeries for guiding volume loading has been shown to improve postoperative outcomes and reduce postoperative hospital length of stay (LOS) [20,21]. Moreover, PPV has demonstrated potential in predicting fluid responsiveness in thoracic surgery patients undergoing “protective” OLV (using low tidal volumes), with satisfactory sensitivity and specificity [22]. However, to the best of our knowledge, the efficacy and safety of PPV-driven fluid management protocols in these patients have not been assessed until now.

The main aim of this study was to investigate the effects of a PPV-guided fluid management protocol on respiratory gas exchange, measured by the PaO_2_/FiO_2_ ratio, in thoracic surgery patients requiring OLV. Additionally, the effects of this protocol on extubation time, the incidence of postoperative pulmonary complications, and LOS were also assessed.

## 2. Materials and Methods

### 2.1. Ethical Statement

This study was approved by the Institutional Ethical Committee (ID 3901), registered on Clincaltrial.gov (identifier: NCT04865874), and conducted in accordance with the ethical standards of the Declaration of Helsinki and its later amendments. All patients signed an informed consent to participate in the study and have their clinical data treated anonymously.

### 2.2. Patients

This study was a prospective, single-center, double-blinded randomized controlled trial (RCT). Patients scheduled for pulmonary lobectomy using the uniportal video-assisted thoracic surgery (VATS) approach were recruited between September 2021 and November 2022 at Fondazione Policlinico Universitario A. Gemelli IRCCS, Rome, Italy. The inclusion criteria of the study were as follows: age ≥ 18 years and American Society of Anesthesiologists physical (ASA) status I-III. The exclusion criteria included the following: patients who refused to sign the informed consent; body mass index (BMI) > 35 kg/m^2^; end-stage kidney disease; cardiac arrhythmias and severe heart dysfunction (New York Heart Association class >III); obstructive sleep apnea syndrome (OSAS); chronic alcoholism; pulmonary resection other than the scheduled lobectomy due to technical or oncological reasons; and conversion to thoracotomy. Furthermore, patients with intraoperative blood losses exceeding 1000 mL and/or those with an OLV duration of less than 60 min were excluded from the study. 

### 2.3. Randomization and Masking

All enrolled patients were allocated in a 1:1 ratio to either the PPV-guided protocol group (PPV group) or the near-zero fluid balance protocol group (control group). Randomization was performed using sequential blocking based on a computer random number generator. Allocation details were kept in sealed envelopes marked with serial numbers. Before the induction of anesthesia, the sealed, numbered, and opaque envelopes containing the treatment assignments were opened by the attending anesthesiologist. To ensure the reliability of data acquisition, patients, clinical researchers collecting data and blood samples, and those conducting postoperative follow-ups were all blinded to group assignments. 

### 2.4. Anesthesia and Monitoring

A standardized anesthesia protocol was implemented for all patients. Intraoperative monitoring included electrocardiogram (ECG), pulse oximetry (SpO_2_), heart rate (HR), non-invasive blood pressure, end-tidal carbon dioxide levels (EtCO_2_), neuromuscular blockade, and bispectral index (BIS). General anesthesia was induced by intravenous injection of Propofol 2 mg/kg, fentanyl 2 mcg/kg, and Rocuronium 0.6 mg/kg was given to facilitate orotracheal intubation. 

After induction of anesthesia, a left double-lumen tube was positioned under bronchoscopic guidance, and a 20-gauge arterial line was inserted into the radial artery of the non-dominant forearm to monitor arterial pressure and collect blood samples for laboratory tests. To evaluate PPV during surgery, the arterial line was connected to the anesthesia machine (Datex-Ohmeda Aisys™ CS2, GE Healthcare, Chicago, IL, USA) designed to detect respiratory variations in the arterial pressure curve, allowing automatic calculation of beat-to-beat pulse pressure. 

PPV was calculated by the machine using the following formula: PPV = 100 × (PPmax − PPmin)/[(PPmax + PPmin)/2]. The mean PPV was automatically calculated over three consecutive periods of eight respiratory cycles, with the median value of these calculations displayed on the multiparameter monitor and updated after each respiratory cycle.

Patients were placed in the lateral decubitus position, and a protective one-lung mechanical ventilation was administered. This included setting the tidal volume (TV) to 4–5 mL/kg, adjusting the respiratory rate to maintain an EtCO2 between 35 and 45 mmHg, and applying a positive end-expiratory pressure (PEEP) of 5 cmH_2_O. The decision to use protective OLV in this study was supported by research from Lee et al. [22] and Minana et al. [23], which demonstrated that PPV can predict fluid responsiveness in patients receiving protective OLV (with a tidal volume of 6 mL/kg) but not in those undergoing conventional ventilation (with a tidal volume of 10 mL/kg). In case of desaturation (SpO_2_< 92%), double-lumen tube position was checked with the bronchoscope. If no issues were found, an alveolar recruitment maneuver was performed according to our center’s standard procedure: the ventilator was switched to pressure-controlled mode, with an inspiratory pressure of 20 cmH_2_O, an inspiratory time of 50%, a respiratory rate of 12 breaths per minute, and PEEP increased by 5 cmH_2_O every 5 breaths until reaching 20 cmH_2_O. After 10 breaths at high pressure, PEEP was reset to 5 cmH_2_O, and ventilation was returned to volume control mode with a TV of 4–5 mL/kg. During surgery, anesthesia was maintained by inhalation of 1–2% sevoflurane and a continuous remifentanil infusion (0.01–0.15 mcg/kg/min) in order to maintain the mean arterial pressure (MAP) between 65 and 90 mmHg and BIS index within 40–60. Neuromuscular monitoring was employed to guide the administration of additional rocuronium boluses during surgery, and sugammadex 2 mg/kg was given to all patients at the end of anesthesia for neuromuscular reversal when spontaneous recovery had occurred up to at least the reappearance of T2. In all patients, the intraoperative inspired O_2_ concentration (FiO_2_) was 100%.

According to Uniportal-VATS technique [24], a single 4–5 cm muscle-sparing incision was performed along the 4th-5th intercostal space on the middle axillary line. A wound protector was used to prevent contamination from tumors or infections, without trocars or rib retractors. A 28Fr chest tube was inserted through the same incision and fixed on the posterior side of it at the end of surgery. In both groups, postoperative analgesia was achieved with paracetamol 1000 mg and ketorolac 30 mg at the end of surgery and by intercostal nerve block performed by the surgeon under direct view with ropivacaine 0.5% 20 mL before chest closure [25]. 

### 2.5. Intervention Protocol

All patients enrolled in the study were allowed to drink clear fluids until two hours before surgery. After general anesthesia induction, the intraoperative basal fluid replacement was achieved in both study groups by continuous infusion of 2 mL/kg/h of Lactate Ringer’s solution, administered through a volumetric infusion pump (Alaris GW, Cardinal Health S.P.A., Milan, Italy).

**Figure 1:**
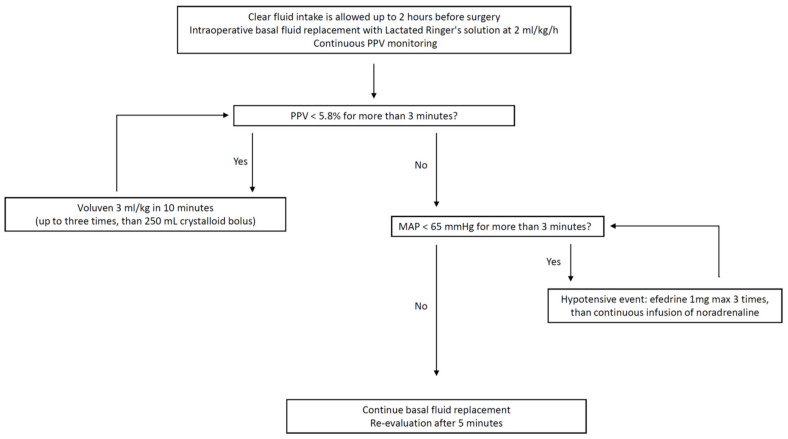
Hemodynamic protocol in the pulse pressure variation (PPV) group. MAP, mean arterial pressure.

For the PPV group, fluid boluses were administered to maintain a continuously measured PPV of 5.8% or less (see Figure 1). This threshold was selected based on findings from a recent RCT by Lee et al., which identified 5.8% as the ideal PPV value to predict a 15% increase in stroke volume in response to a fluid challenge in patients undergoing OLV [22].

If the PPV exceeded the 5.8% threshold, a 3 mL/kg colloid bolus (hydroxyethyl starch 6% 130/0.4, Voluven, Fresenius Kabi, Germany) was given over 10 min. This dose could be repeated up to three times if the PPV remained above 5.8%. If the PPV still exceeded 5.8% after three doses, or if it increased above the threshold again, hemodynamic support was then provided with crystalloid boluses (250 mL Lactate Ringer solution). Colloids were chosen as the first-line treatment due to their favorable properties for plasma expansion and intravascular retention time. We selected hydroxyethyl starch 130/0.4 among colloids, as it is considered to have a lower risk of renal injury and coagulopathy compared to older formulations and was the most commonly used starch in Italy at the time. Albumin was avoided because it is expensive, being a human product, and is sometimes in short supply. However, due to cautions from regulatory authorities in both Europe [26] and the United States [27] regarding the use of hydroxyethyl starch solutions in patients with sepsis, burn injuries, or critical illness, we decided to limit the maximum dose of hydroxyethyl starch in this study to 9 mL/kg as a precaution. This decision was made even though patients undergoing major surgery are generally less fragile than critical care patients. 

**Figure 2:**
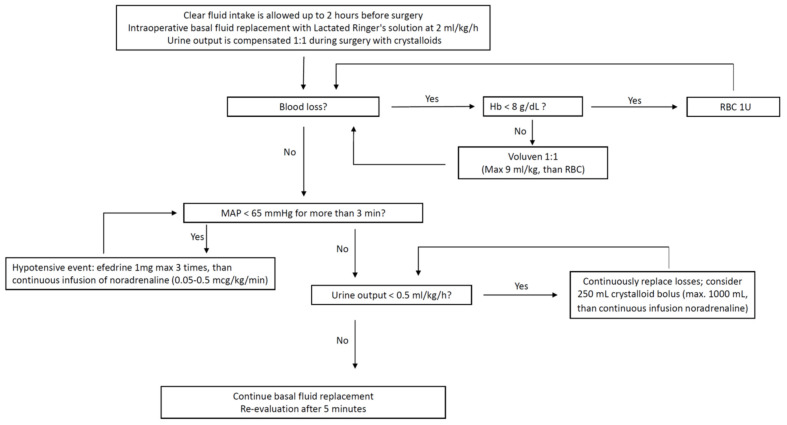
Hemodynamic protocol in the near-zero group. MAP, mean arterial pressure; Hb, hemoglobin; RBC, red blood cells.

For the near-zero group (control group), urine output was compensated 1:1 during surgery with Lactate Ringer solution, and hemodynamic management was performed according to a pre-established protocol (see Figure 2) to achieve a minimum MAP of 65 mmHg and urinary output higher than 0.5 mL/kg/hr. Interventions included fluid resuscitation with Lactate Ringer solution or colloids (Voluven), administration of vasopressors (etilefrine or noradrenaline), and red blood cells (RBC) transfusion. Crystalloid boluses (Lactate Ringer 250 mL) were allowed up to a total of 1000 mL. Etilefrine boluses of 1 mg were administered up to three times if MAP fell below 65 mmHg. These episodes were documented as hypotensive events and analyzed. If MAP remained below 65 mmHg after three consecutive hypotensive events, a continuous infusion of noradrenaline (0.05–1 mcg/kg/min) was initiated.

The respective hemodynamic protocols for both groups were maintained until the end of the surgery. Immediately after extubation, patients were transferred to the post-anesthetic recovery room (PAR) and monitored for approximately three hours. Before being moved to the surgical ward at the end of this monitoring period, all patients underwent a chest radiograph. The primary clinical and surgical variables recorded prospectively for each patient are reported in Table 1. Additional perioperative variables recorded were as follows: surgery duration, OLV duration, total volume of crystalloids and colloids administered during surgery, intraoperative blood loss, urine output, total number of etilefrine doses, use of noradrenaline, and time to extubation, which was measured as the interval between the discontinuation of all anesthetics immediately after skin closure and the removal of the endotracheal tube once adequate spontaneous breathing was confirmed. Lastly, postoperative pain, PONV, PPCs within the first three days after surgery, and LOS were also assessed. Postoperative pain was assessed using the Numeric Pain Rating Scale (NPRS), a scale ranging from 0 to 10 (where 0 represents no pain and 10 corresponds to the worst imaginable pain), two hours after extubation while at rest. PPCs included ARDS, reintubation, pneumonia, need for bedside bronchoscopy, atelectasis, pulmonary embolism, prolonged air leak, and failure to expand during postoperative hospitalization. Postoperative pneumonia was defined as a new pulmonary infiltrate on a chest x-ray with leucocytosis and fever (ear temperature > 37.5 °C) [28,29]. Atelectasis was diagnosed by chest radiograph documentation, and pulmonary embolism was diagnosed by pulmonary computed tomography angiography [28,29]. Prolonged air leak was defined as leak > 5 days. In the case of failure to expand the diagnosis was made if the inability of the remaining lung to fill the pleural cavity with or without air leak was seen on chest x-ray. LOS was defined as the time elapsed between the day of surgery and discharge.

### 2.6. Primary and Secondary Outcomes 

The primary outcome was the PaO_2_/FiO_2_ ratio calculated fifteen minutes after extubation. Secondary outcomes were time to extubation, incidence of PPCs in the first three postoperative days, and LOS. 

### 2.7. Statistical Analysis

GPower* software (version 3.1.9.7) was used for a priori power analysis. Based on previous evidence [16,17,18], a 20% increase in the PaO_2_/FiO_2_ ratio in the PPV group (400 vs. 320 with a standard deviation of 120) was hypothesized. A sample of 80 patients (40 per group) was required to achieve a power of 90% with α = 0.05 (effect size d: 0.66). Considering a drop-out rate of 17%, a minimum sample size of 94 patients was calculated. All data were displayed as medians (standard deviation) or numbers and tabulated descriptively by the study group. After verifying the normal distribution with the Kolmogorov–Smirnov test, Student’s *t*-test was used to analyze continuous variables. Categorical variables were analyzed by chi-square test. A value of *p* < 0.05 was considered statistically significant.

## 3. Results

**Figure 3:**
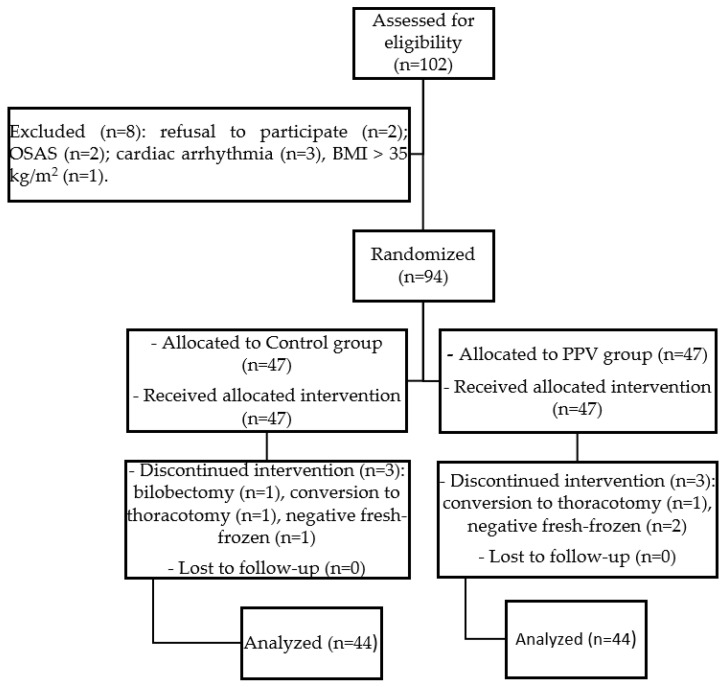
Study flow diagram. OSAS, obstructive sleep apnea syndrome; BMI, body mass index.

One hundred and two patients were considered eligible for this study. Eight patients were excluded for the following reasons: refusal to participate (*n* = 2); OSAS (*n* = 2) and cardiac arrhythmia (*n* = 3), BMI > 35 kg/m^2^ (*n* = 1). Ninety-four were randomly assigned to two groups. Six patients were excluded due to conversion from VATS to thoracotomy for technical reasons (*n* = 2), and when surgery was limited to wedge resection instead of the scheduled lobectomy for the negative fresh-frozen (*n* = 3) or to bilobectomy (*n* = 1). Data from 88 patients were finally analyzed (Figure 3). 

No differences in preoperative characteristics and comorbidities between the two groups were noted (Table 1). 

The primary outcome (PaO_2_/FiO_2_) did not differ between the two groups (364.48 ± 38.06 vs 359.21 ± 36.95; t = 0.66; *p* = 0.51). Importantly, the patients from the PPV group received more crystalloids (*p* = 0.01) and colloids (*p* = 0.002) than in the near-zero group.

**Figure 4:**
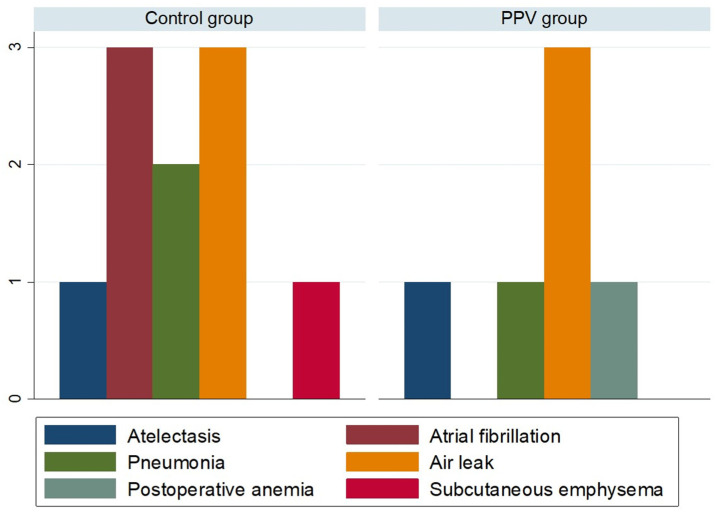
Number of patients with postoperative pulmonary complications in the two groups. PPV, pulse pressure variation.

The number and type of PPCs did not differ between groups (Table 2, Figure 4): subcutaneous emphysema (χ^2^ = 0.01; *p*= 0.31); postoperative anemia (χ^2^ = 1.01; *p* = 0.31); air leak (χ^2^ = 0.00; *p*= 1.00); pneumonia (χ^2^ = 0.34; *p* = 0.56); and atrial fibrillation (χ^2^ = 3.11; *p* = 0.08; atelectasis (χ^2^ = 0.00; *p* = 1.00). None of the patients were transfused.

The extubation time, LOS, and incidence of PONV did not differ between the two study groups (Table 2).

## 4. Discussion

The results of this study indicate that implementing a PPV-guided fluid management protocol in thoracic surgery requiring one-lung ventilation, while feasible and safe, does not enhance respiratory gas exchange during the perioperative period as measured by the PaO_2_/FiO_2_ ratio nor reduce respiratory complications within the first 3 days after surgery. Additionally, this approach does not reduce extubation time or the incidence of PONV and LOS, and leads to a higher fluid administration compared to a conventional near-zero fluid balance protocol. These findings do not definitively rule out the possibility that PPV-guided fluid management in thoracic surgery may provide similar advantages to those seen in cardiac and abdominal surgeries [20,30,31]. However, according to our results, this strategy does not seem to offer clinical benefits.

In thoracic surgery, the potential benefits and drawbacks of fluid management guided by dynamic indices of preload are still under-evaluated. Specifically, two main questions remain unanswered: firstly, whether preload functional parameters can accurately predict fluid responsiveness in patients undergoing thoracic surgery requiring OLV; and secondly, whether PPV- or SVV-driven protocols can improve clinical outcomes in these patients.

Regarding the first point, existing studies on dynamic preload indices in thoracic surgery often show conflicting results. For instance, a recent RCT by Jeong et al. found that SVV and PPV do not accurately predict fluid responsiveness in patients undergoing thoracic surgery with OLV [31]. These findings have been supported by more recent studies [32]. However, Fu et al. reported that dynamic preload indices could predict fluid responsiveness in such patients with acceptable accuracy, suggesting that PPV may be superior to SVV during protective OLV [33].

SVV and PPV are derived from the cyclical changes in stroke volume and pulse pressure caused by intrathoracic pressure fluctuations induced by positive pressure mechanical ventilation. As these variations increase in the case of hypovolemia, dynamic preload indices have been used to assess fluid responsiveness in various surgeries, often showing greater sensitivity and specificity than static preload parameters. However, PPV and SVV also depend on numerous respiratory and cardiovascular factors (e.g., TV, PEEP, thoraco-pulmonary compliance, HR, rhythm, systolic function, afterload, arterial compliance), which can vary significantly in thoracic patients due to lateral decubitus positioning, chest opening, and other surgical conditions. These factors potentially reduce their accuracy in predicting fluid responsiveness in this setting.

However, current data are insufficient to draw definitive conclusions, as published studies are often heterogeneous and conflicting. The high I^2^ values highlight the limitations of meta-analysis techniques in deriving conclusive results from the published literature, especially regarding PPV [34,35]. Consequently, since the question is not conclusively answered, the reliability of dynamic preload indices to identify “responders” patients to volume loading in the context of thoracic surgery and OLV remains unclear.

The aim of this study was not to evaluate the accuracy of dynamic preload indices in predicting fluid responsiveness in patients undergoing thoracic surgery requiring OLV. Instead, we explored the effects of a PPV-guided fluid protocol on respiratory gas exchange and the rate of postoperative pulmonary complications in this surgical context, as these aspects had not been comprehensively assessed in clinical practice.

In a good quality RCT, Lee et al. demonstrated that PPV could predict fluid responsiveness in patients undergoing protective OLV (TV = 6 mL/kg), but not in those undergoing conventional ventilation (TV = 10 mL/kg) [22]. These results were more recently confirmed by Minana et al. [23], leading us to select a TV of 6 mL/kg for our study. Furthermore, based on Lee et al.’s identification of an optimal PPV threshold value of 5.8% to predict a 15% increase in stroke volume in response to a fluid challenge [22], we adopted this threshold for fluid administration in our protocol.

In the PPV-group, fluid administration aimed at maintaining a PPV value of 5.8% during surgery resulted in greater use of both crystalloids (1145 ± 470.21 vs 890 ± 459.31, *p* = 0.01) and colloids (162.50 ± 278.31 vs 18.18 ± 94.68, *p* = 0.002) compared with the near zero-group. However, this increased fluid volume did not affect the PaO_2_/FiO_2_ ratio, extubation time, or incidence of PPCs. These results differ from previous studies by Xu [17] and Zhang [16], which reported that SVV-targeted fluid protocols reduced fluid volumes, improved PaO_2_/FiO_2_ ratios, decreased extubation times, and lowered rates of nausea, vomiting, and PPCs.

Several factors may explain these findings. Firstly, the 5.8% threshold for fluid loading in this study was significantly lower than the 11% and 13% thresholds used in Zhang’s and Xu’s studies, respectively. This likely led to a higher volume of fluids being administered in the PPV-guided group compared to the two aforementioned studies. Additionally, the total amount of crystalloids administered to patients in the intervention group in this study (1145 ± 470 mL) was higher than in Zhang’s study (625 ± 100 mL) and Xu’s study (490 ± 194 mL) [16,17]. It is well recognized that only one-fifth of intravenously infused crystalloids remains within the intravascular space compared to colloids [36]. Therefore, the larger volume of crystalloids administered in the PPV-guided group may have increased pulmonary extravascular fluid content, potentially leading to the development of interstitial or alveolar edema and a more pronounced—though not harmful—deterioration in pulmonary function.

Furthermore, the control groups in Zhang’s and Xu’s studies [16,17] followed more aggressive fluid protocols compared to this study. Zhang’s study employed a basal continuous infusion rate of 8 mL/kg/h [16], and Xu’s study used 4 mL/kg/h [17], which are four times and twice the rate used in this study (2 mL/kg/h), respectively. This likely led to excessive fluid administration, which could further explain why the authors observed a decrease in total fluid volume in patients monitored using dynamic preload indices. Indeed, patients in the control groups may have received a significantly greater total amount of fluids compared to those in the intervention groups.

Therefore, the results of the few existing studies on the use of dynamic preload indices for fluid management during thoracic surgery requiring OLV could be at least partly influenced by the type of fluid regimen used in the control group. These regimens are often described by authors as “restrictive” or “moderate”, but these definitions are subjective. The recommended practice of maintaining a dry lung through minimized fluid administration of 2–5 mL/kg/h (especially in VATS/RATS procedures) was not consistently followed [37]. This underscores the need for a more objective approach to fluid therapy, particularly for the most vulnerable patients, in future studies on this topic.

This study has several limitations. Firstly, it was conducted at a single center, which may limit the generalizability of our results. Secondly, the study only included patients who underwent thoracoscopic lung procedures, so the findings cannot be applied to those undergoing open and non-pulmonary thoracic surgery. Thirdly, the extravascular fluid content in the lungs could have been measured using a sensitive parameter, such as the extravascular lung water index obtained through thermodilution. Fourthly, the inflammatory response, which is crucial in the development of lung injury during OLV, could not be measured because serum cytokine levels, including tumor necrosis factor-α, interleukin (IL)-6, and IL-10, were not recorded during the perioperative period. Lastly, the incidence of acute kidney injury and long-term outcomes for the study population were not reported.

Despite these limitations, the study has several strengths. Firstly, it is the first to evaluate the impact of a PPV-guided fluid management protocol on respiratory gas exchange and the rate of postoperative pulmonary complications in thoracic surgery requiring OLV. Secondly, it was a prospective, randomized trial with a sample size calculated based on the primary outcome. Thirdly, to minimize bias, all patients underwent the same surgical procedure (pulmonary lobectomy) performed by experienced surgeons in the same manner (Uniportal-VATS). Fourthly, perioperative pain management was standardized, and no patients received thoracic epidurals. This is important because volume responsiveness can be affected by decreased intravascular tone, especially when local epidural anesthetics are used during surgery.

## 5. Conclusions

In conclusion, PPV-guided fluid management in thoracic surgery requiring OLV does not appear to improve pulmonary gas exchange or clinical outcomes and leads to increased fluid administration compared to a conventional near-zero fluid balance protocol. Nevertheless, the concept of GDT in thoracic surgery is still in its infancy, and current evidence is insufficient to draw firm conclusions on the topic. Further high-quality studies are urgently needed to determine the safety, efficacy, optimal timing of fluid application, type of hemodynamic monitoring, and the most suitable type of fluid for its use in thoracic surgery and OLV. Although the effects of GDT in thoracic surgery requiring OLV may have been overestimated in recent years, GDT likely remains a more objective approach than empirical methods. Therefore, its effects must be rigorously investigated and thoroughly evaluated before considering alternative approaches, particularly in more severe patients, where correct decision-making is crucial.

## Figures and Tables

**Table 1:** Characteristics and comorbidities of patients from the two groups. Values are means (standard deviation) or numbers (%).

	Near-Zero Group (*n* = 44)	PPV Group (*n* = 44)	t or χ^2^	*p*
Age, years	68.93 (8.71)	66.82 (11.53)	0.97	0.33
BMI	25.20 (3.88)	26.00 (4.11)	−0.93	0.35
NYHA class (I/II/III)	30/13/1	27/17/0	1.69	0.43
ASA score, II/III	30/14	29/15	0.05	0.82
AH (Y/N)	14/30	31/13	0.05	0.82
IHD (Y/N)	3/41	2/42	0.21	0.64
DM II (Y/N)	6/38	5/39	0.10	0.75
Bronchial Asthma (Y/N)	0/44	2/42	2.05	0.15
COPD (Y/N)	23/21	24/20	0.04	0.83
VHD (Y/N)	2/42	1/43	0.34	0.56
CKD (Y/N)	2/42	2/42	0.00	1.00
CV (Y/N)	1/43	0/44	1.01	0.31
PVD	3/41	1/43	1.05	0.31
FVC	3.49 (0.93)	3.33 (0.81)	0.85	0.40
FEV1	2.58 (0.69)	2.50 (0.68)	0.54	0.59
FEV1/FVC (%)	74.42 (7.66)	75.16 (8.08)	−0.45	0.66
DLCO (%)	78.80 (7.07)	79.62 (7.57)	−0.52	0.60
Hemoglobin, gr/dL	13.59 (1.47)	13.65 (1.69)	−0.20	0.84
Creatinine, mg/dL	1.69 (3.79)	0.89 (0.21)	1.39	0.17
BUN, mg/dL	18.55 (7.21)	18.39 (5.47)	0.11	0.91

Abbreviations: PPV, pulse pressure variation; BMI, body mass index; Y/N, Yes/No; AH, arterial hypertension; IHD, ischemic heart disease; DM, diabetes mellitus; COPD, chronic obstructive pulmonary disease; ASA, American Society of Anesthesiologists; VHD, valvular heart disease; CKD, chronic kidney disease; CV, cerebral vasculopathy; PVD, peripheral vascular disease; FVC, forced vital capacity; FEV1, forced expiratory volume in 1 s; DLCO, diffusing capacity of the lungs for carbon monoxide; BUN, blood urea nitrogen.

**Table 2:** Intraoperative and postoperative variables in the two groups. Values are means (standard deviation) or numbers.

	Near-Zero Group (*n* = 44)	PPV Group (*n* = 44)	t or χ^2^	*p*
Duration of surgery, min	154.16 ± 44.25	170.82 ± 46.38	−1.72	0.09
Duration of OLV, min	121.66 ± 38.73	137.29 ± 41.79	−1.82	0.07
Blood loss, mL	52.50 ± 104.97	47.27	0.27	0.79
Crystalloids, mL	890 ± 459.31	1145 ± 470.21	−2.57	0.01
Colloids, mL	18.18 ± 94.68	162.50 ± 278.31	−3.26	0.002
RBC units, *n*	0	0	-	-
Urine output, mL	324.54 ± 222.63	357.95 ± 233.14	−0.69	0.49
Hypotensive events	2.14 ± 2.16	1.48 ± 1.87	1.53	0.13
Noradrenaline use, Y/N	8/36	7/37	0.08	0.78
Extubation time, min	12.70 ± 4.77	12.41 ± 4.64	0.29	0.77
PONV, Y/N	1/43	2/42	0.34	0.56
NPRS	1.89 ± 1.79	1.41 ± 1.47	1.35	0.17
PPCs, Y/N	9/35	7/37	0.31	0.58
LOS, days	4.25 ± 1.83	4.25 ± 1.59	0.00	1.00

Abbreviations: PPV, pulse pressure variation; Y/N, Yes/No; OLV, one-lung ventilation; NPRS, numeric pain rating scale; RBC, packed red blood cells; PONV, postoperative nausea and vomiting; PPCs, postoperative pulmonary complications; LOS, length of hospital stay.

## Data Availability

The data presented in this study are available on request from the corresponding author. The data are not publicly available according to the privacy policy.
